# (*E*)-*N*′-(2-Hy­droxy-3,5-diiodo­benzyl­idene)nicotinohydrazide acetonitrile monosolvate

**DOI:** 10.1107/S1600536811020770

**Published:** 2011-06-11

**Authors:** Shan-Shan Sun, Shi-Yong Liu, Ting-Ting Zheng, Xiao-Ling Wang

**Affiliations:** a College of Chemistry and Pharmacy, Taizhou University, Taizhou Zhejiang 317000, People’s Republic of China; b Department of Chemistry, Liaoning Normal University, Dalian 116029, People’s Republic of China

## Abstract

In the hydrazone molecule of the title compound, C_13_H_9_I_2_N_3_O_2_·CH_3_CN, the aromatic rings form a dihedral angle of 9.4 (3)°. In the crystal structure, inter­molecular I⋯N inter­actions [3.099 (4) Å] link hydrogen-bonded aggregates of the hydrozone and solvent molecules related by translation along the *b* axis into chains. An intramolecular O—H⋯N hydrogen bond forms an *S*(6) ring.

## Related literature

For the crystal structures of hydrazones recently reported by us, see: Liu & You (2010*a* ▶,*b* ▶,*c* ▶); Liu & Wang (2010*a* ▶,*b* ▶; 2011 ▶).
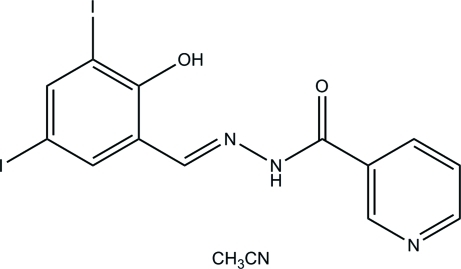


## Experimental

### 

#### Crystal data

C_13_H_9_I_2_N_3_O_2_·C_2_H_3_N
*M* *_r_* = 534.09
Monoclinic, 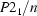

*a* = 11.1347 (13) Å
*b* = 13.3721 (16) Å
*c* = 11.9999 (15) Å
β = 104.083 (6)°
*V* = 1733.0 (4) Å^3^
*Z* = 4
Mo *K*α radiation
μ = 3.64 mm^−1^
*T* = 298 K
0.23 × 0.22 × 0.20 mm

#### Data collection

Bruker SMART CCD area-detector diffractometer
Absorption correction: multi-scan (*SADABS*; Bruker, 2001 ▶) *T*_min_ = 0.488, *T*_max_ = 0.529
10053 measured reflections
3546 independent reflections
2730 reflections with *I* > 2σ(*I*)
*R*_int_ = 0.031

#### Refinement

*R*[*F* ^2^ > 2σ(*F* ^2^)] = 0.028
*wR*(*F* ^2^) = 0.062
*S* = 1.01
3546 reflections
213 parameters
1 restraint
H atoms treated by a mixture of independent and constrained refinement
Δρ_max_ = 0.41 e Å^−3^
Δρ_min_ = −0.37 e Å^−3^

### 

Data collection: *SMART* (Bruker, 2007 ▶); cell refinement: *SAINT* (Bruker, 2007 ▶); data reduction: *SAINT*; program(s) used to solve structure: *SHELXTL* (Sheldrick, 2008 ▶); program(s) used to refine structure: *SHELXTL*; molecular graphics: *SHELXTL*; software used to prepare material for publication: *SHELXTL*.

## Supplementary Material

Crystal structure: contains datablock(s) global, I. DOI: 10.1107/S1600536811020770/cv5102sup1.cif

Structure factors: contains datablock(s) I. DOI: 10.1107/S1600536811020770/cv5102Isup2.hkl

Supplementary material file. DOI: 10.1107/S1600536811020770/cv5102Isup3.cml

Additional supplementary materials:  crystallographic information; 3D view; checkCIF report

## Figures and Tables

**Table 1:** Hydrogen-bond geometry (Å, °)

*D*—H⋯*A*	*D*—H	H⋯*A*	*D*⋯*A*	*D*—H⋯*A*
O1—H1⋯N1	0.82	1.86	2.584 (4)	147
N2—H2⋯N4	0.90 (1)	2.22 (2)	3.076 (5)	160 (4)

**Fig. 1.**
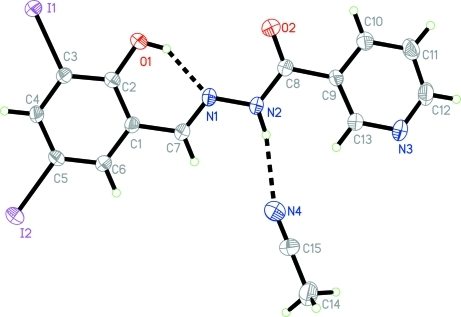
The molecular structure of (I). Displacement ellipsoids are drawn at the 30% probability level. H atoms are shown as spheres of arbitrary radius and the hydrogen bonds are drawn as dashed lines.

## supplementary crystallographic information

### Comment

In continuation of our structural studies of hydrazone derivatives (Liu & You, 2010*a*,*b*,*c*; Liu & Wang, 2010*a*,*b*; 2011), we present here the title compound (I).

In the asymmetric part of (I) (Fig. 1), the acetonitrile molecule is linked to the hydrazone molecule through intermolecular N–H···N hydrogen bond (Table 1). An intramolecular O—H···N hydrogen bond (Table 1) affects the molecular conformation - the dihedral angle between the C1–C6 benzene ring and the C9–C13/N3 pyridine ring is 9.4 (3)°.

In the crystal structure, there is I1···N3(x, -1 + y, z) [3.099 (4) Å] interaction, which link the molecules into chains along *b* axis.

### Experimental

The title compound was prepared by the condensation reaction of 2-hydroxy-3,5-diiodobenzaldehyde (1.0 mmol, 0.374 g) and nicotinohydrazide (1.0 mmol, 0.137 g) in acetonitrile (50 ml) at ambient temperature. Colourless block-shaped single crystals suitable for X-ray structural determination were obtained by slow evaporation of the solution for a few days.

### Refinement

H2 was located from a difference Fourier map and refined isotropically, with the N—H distance restrained to 0.90 (1) Å. The remaining H atoms were positioned geometrically and constrained to ride on their parent atoms, with C—H distances of 0.93–0.96 Å, O—H distance of 0.82 Å, and with *U*_iso_(H) = 1.2–1.5*U*_eq_ of the parent atom.

### Crystal data

**Table:**

C_13_H_9_I_2_N_3_O_2_·C_2_H_3_N	*F*(000) = 1008
*M**_r_* = 534.09	*D*_x_ = 2.047 Mg m^−^^3^
Monoclinic, *P*2_1_/*n*	Mo *K*α radiation, λ = 0.71073 Å
*a* = 11.1347 (13) Å	Cell parameters from 2928 reflections
*b* = 13.3721 (16) Å	θ = 2.4–26.0°
*c* = 11.9999 (15) Å	µ = 3.64 mm^−^^1^
β = 104.083 (6)°	*T* = 298 K
*V* = 1733.0 (4) Å^3^	Block, colourless
*Z* = 4	0.23 × 0.22 × 0.20 mm

### Data collection

**Table:**

Bruker SMART CCD area-detector diffractometer	3546 independent reflections
Radiation source: fine-focus sealed tube	2730 reflections with *I* > 2σ(*I*)
graphite	*R*_int_ = 0.031
ω scans	θ_max_ = 26.4°, θ_min_ = 2.2°
Absorption correction: multi-scan (*SADABS*; Bruker, 2001)	*h* = −13→13
*T*_min_ = 0.488, *T*_max_ = 0.529	*k* = −15→16
10053 measured reflections	*l* = −9→14

### Refinement

**Table:**

Refinement on *F*^2^	Primary atom site location: structure-invariant direct methods
Least-squares matrix: full	Secondary atom site location: difference Fourier map
*R*[*F*^2^ > 2σ(*F*^2^)] = 0.028	Hydrogen site location: inferred from neighbouring sites
*wR*(*F*^2^) = 0.062	H atoms treated by a mixture of independent and constrained refinement
*S* = 1.01	*w* = 1/[σ^2^(*F*_o_^2^) + (0.0273*P*)^2^] where *P* = (*F*_o_^2^ + 2*F*_c_^2^)/3
3546 reflections	(Δ/σ)_max_ < 0.001
213 parameters	Δρ_max_ = 0.41 e Å^−^^3^
1 restraint	Δρ_min_ = −0.37 e Å^−^^3^

### Special details

**Table:**

Geometry. All esds (except the esd in the dihedral angle between two l.s. planes) are estimated using the full covariance matrix. The cell esds are taken into account individually in the estimation of esds in distances, angles and torsion angles; correlations between esds in cell parameters are only used when they are defined by crystal symmetry. An approximate (isotropic) treatment of cell esds is used for estimating esds involving l.s. planes.
Refinement. Refinement of F^2^ against ALL reflections. The weighted R-factor wR and goodness of fit S are based on F^2^, conventional R-factors R are based on F, with F set to zero for negative F^2^. The threshold expression of F^2^ > 2sigma(F^2^) is used only for calculating R-factors(gt) etc. and is not relevant to the choice of reflections for refinement. R-factors based on F^2^ are statistically about twice as large as those based on F, and R- factors based on ALL data will be even larger.

### Fractional atomic coordinates and isotropic or equivalent isotropic displacement parameters (Å^2^)

**Table:**

	*x*	*y*	*z*	*U*_iso_*/*U*_eq_	
I1	0.58225 (2)	0.137781 (18)	0.14099 (2)	0.04871 (9)	
I2	0.97606 (3)	0.30924 (2)	−0.06800 (2)	0.05637 (10)	
N1	0.5759 (3)	0.5602 (2)	0.1531 (3)	0.0441 (8)	
N2	0.5445 (3)	0.6581 (2)	0.1681 (3)	0.0461 (8)	
N3	0.4412 (4)	0.9562 (2)	0.2073 (3)	0.0625 (10)	
N4	0.7239 (4)	0.8004 (3)	0.0881 (4)	0.0773 (12)	
O1	0.5386 (3)	0.37009 (19)	0.1633 (3)	0.0524 (7)	
H1	0.5268	0.4298	0.1716	0.079*	
O2	0.4105 (3)	0.6067 (2)	0.2699 (3)	0.0652 (8)	
C1	0.6974 (3)	0.4418 (2)	0.0834 (3)	0.0375 (8)	
C2	0.6354 (3)	0.3585 (3)	0.1150 (3)	0.0394 (8)	
C3	0.6742 (3)	0.2631 (3)	0.0952 (3)	0.0399 (9)	
C4	0.7714 (3)	0.2493 (3)	0.0444 (3)	0.0413 (9)	
H4	0.7969	0.1850	0.0318	0.050*	
C5	0.8309 (3)	0.3311 (3)	0.0123 (3)	0.0399 (9)	
C6	0.7952 (3)	0.4261 (3)	0.0316 (3)	0.0418 (9)	
H6	0.8364	0.4805	0.0101	0.050*	
C7	0.6612 (3)	0.5441 (3)	0.1014 (3)	0.0440 (9)	
H7	0.7004	0.5975	0.0752	0.053*	
C8	0.4560 (3)	0.6742 (3)	0.2269 (3)	0.0428 (9)	
C9	0.4165 (3)	0.7803 (3)	0.2325 (3)	0.0392 (8)	
C10	0.3099 (4)	0.7986 (3)	0.2676 (4)	0.0538 (10)	
H10	0.2664	0.7459	0.2898	0.065*	
C11	0.2676 (4)	0.8956 (3)	0.2697 (4)	0.0628 (12)	
H11	0.1944	0.9095	0.2910	0.075*	
C12	0.3370 (4)	0.9702 (3)	0.2396 (4)	0.0613 (12)	
H12	0.3092	1.0355	0.2419	0.074*	
C13	0.4789 (4)	0.8619 (3)	0.2048 (3)	0.0518 (10)	
H13	0.5522	0.8504	0.1828	0.062*	
C14	0.8500 (5)	0.9474 (3)	0.0420 (4)	0.0834 (16)	
H14A	0.8874	0.9288	−0.0191	0.125*	
H14B	0.9135	0.9650	0.1088	0.125*	
H14C	0.7963	1.0037	0.0184	0.125*	
C15	0.7793 (4)	0.8646 (3)	0.0688 (4)	0.0595 (11)	
H2	0.580 (4)	0.707 (2)	0.135 (3)	0.080*	

### Atomic displacement parameters (Å^2^)

**Table:**

	*U*^11^	*U*^22^	*U*^33^	*U*^12^	*U*^13^	*U*^23^
I1	0.05615 (17)	0.03602 (14)	0.06087 (19)	−0.00160 (11)	0.02760 (13)	0.00167 (12)
I2	0.05261 (17)	0.05701 (18)	0.0699 (2)	0.00518 (13)	0.03507 (14)	0.00061 (14)
N1	0.0471 (19)	0.0306 (16)	0.057 (2)	0.0023 (13)	0.0177 (16)	−0.0047 (14)
N2	0.052 (2)	0.0276 (17)	0.064 (2)	0.0024 (14)	0.0242 (17)	−0.0030 (15)
N3	0.075 (3)	0.0336 (18)	0.085 (3)	0.0053 (17)	0.032 (2)	−0.0059 (18)
N4	0.059 (3)	0.066 (3)	0.115 (4)	0.001 (2)	0.038 (2)	0.005 (3)
O1	0.0511 (17)	0.0445 (15)	0.0723 (19)	0.0018 (13)	0.0359 (14)	−0.0019 (15)
O2	0.072 (2)	0.0430 (16)	0.094 (2)	−0.0027 (15)	0.0471 (18)	0.0030 (16)
C1	0.044 (2)	0.0317 (19)	0.039 (2)	−0.0001 (15)	0.0135 (16)	−0.0021 (15)
C2	0.040 (2)	0.041 (2)	0.040 (2)	0.0008 (16)	0.0158 (17)	−0.0008 (17)
C3	0.048 (2)	0.0331 (19)	0.042 (2)	0.0001 (16)	0.0179 (17)	0.0019 (16)
C4	0.049 (2)	0.0339 (19)	0.046 (2)	0.0074 (17)	0.0198 (18)	−0.0004 (17)
C5	0.038 (2)	0.043 (2)	0.042 (2)	0.0032 (16)	0.0184 (17)	−0.0005 (17)
C6	0.046 (2)	0.0345 (19)	0.048 (2)	−0.0018 (16)	0.0172 (18)	0.0011 (17)
C7	0.050 (2)	0.0325 (19)	0.053 (2)	0.0013 (16)	0.0198 (19)	0.0016 (17)
C8	0.047 (2)	0.035 (2)	0.049 (2)	−0.0027 (17)	0.0179 (19)	−0.0044 (17)
C9	0.042 (2)	0.0356 (19)	0.044 (2)	−0.0013 (15)	0.0177 (17)	−0.0080 (17)
C10	0.050 (2)	0.047 (2)	0.069 (3)	−0.0041 (19)	0.023 (2)	−0.011 (2)
C11	0.043 (2)	0.061 (3)	0.088 (3)	0.005 (2)	0.024 (2)	−0.019 (3)
C12	0.064 (3)	0.044 (2)	0.076 (3)	0.008 (2)	0.017 (2)	−0.015 (2)
C13	0.056 (3)	0.037 (2)	0.070 (3)	−0.0006 (18)	0.031 (2)	−0.005 (2)
C14	0.114 (4)	0.052 (3)	0.098 (4)	0.001 (3)	0.052 (3)	0.009 (3)
C15	0.060 (3)	0.054 (3)	0.072 (3)	0.012 (2)	0.030 (2)	0.003 (2)

### Geometric parameters (Å, °)

**Table:**

I1—C3	2.106 (3)	C4—H4	0.9300
I2—C5	2.094 (3)	C5—C6	1.367 (5)
N1—C7	1.273 (4)	C6—H6	0.9300
N1—N2	1.377 (4)	C7—H7	0.9300
N2—C8	1.362 (5)	C8—C9	1.492 (5)
N2—H2	0.897 (10)	C9—C10	1.375 (5)
N3—C12	1.323 (5)	C9—C13	1.377 (5)
N3—C13	1.332 (5)	C10—C11	1.382 (5)
N4—C15	1.114 (5)	C10—H10	0.9300
O1—C2	1.351 (4)	C11—C12	1.364 (6)
O1—H1	0.8200	C11—H11	0.9300
O2—C8	1.210 (4)	C12—H12	0.9300
C1—C6	1.395 (5)	C13—H13	0.9300
C1—C2	1.411 (5)	C14—C15	1.440 (6)
C1—C7	1.457 (5)	C14—H14A	0.9600
C2—C3	1.385 (5)	C14—H14B	0.9600
C3—C4	1.377 (5)	C14—H14C	0.9600
C4—C5	1.382 (5)		
			
C7—N1—N2	117.9 (3)	C1—C7—H7	120.1
C8—N2—N1	117.2 (3)	O2—C8—N2	122.3 (3)
C8—N2—H2	124 (3)	O2—C8—C9	122.1 (3)
N1—N2—H2	118 (3)	N2—C8—C9	115.6 (3)
C12—N3—C13	116.4 (4)	C10—C9—C13	117.2 (3)
C2—O1—H1	109.5	C10—C9—C8	118.0 (3)
C6—C1—C2	119.1 (3)	C13—C9—C8	124.8 (3)
C6—C1—C7	118.8 (3)	C9—C10—C11	119.8 (4)
C2—C1—C7	122.1 (3)	C9—C10—H10	120.1
O1—C2—C3	119.6 (3)	C11—C10—H10	120.1
O1—C2—C1	121.2 (3)	C12—C11—C10	117.6 (4)
C3—C2—C1	119.2 (3)	C12—C11—H11	121.2
C4—C3—C2	120.7 (3)	C10—C11—H11	121.2
C4—C3—I1	119.6 (3)	N3—C12—C11	124.7 (4)
C2—C3—I1	119.7 (3)	N3—C12—H12	117.7
C3—C4—C5	120.0 (3)	C11—C12—H12	117.7
C3—C4—H4	120.0	N3—C13—C9	124.4 (4)
C5—C4—H4	120.0	N3—C13—H13	117.8
C6—C5—C4	120.6 (3)	C9—C13—H13	117.8
C6—C5—I2	119.8 (3)	C15—C14—H14A	109.5
C4—C5—I2	119.6 (3)	C15—C14—H14B	109.5
C5—C6—C1	120.5 (3)	H14A—C14—H14B	109.5
C5—C6—H6	119.8	C15—C14—H14C	109.5
C1—C6—H6	119.8	H14A—C14—H14C	109.5
N1—C7—C1	119.8 (3)	H14B—C14—H14C	109.5
N1—C7—H7	120.1	N4—C15—C14	179.2 (6)

### Hydrogen-bond geometry (Å, °)

**Table:**

*D*—H···*A*	*D*—H	H···*A*	*D*···*A*	*D*—H···*A*
O1—H1···N1	0.82	1.86	2.584 (4)	147.
N2—H2···N4	0.90 (1)	2.22 (2)	3.076 (5)	160 (4)
